# Correction: Gemoll et al. Protein Profiling of Serum Extracellular Vesicles Reveals Qualitative and Quantitative Differences after Differential Ultracentrifugation and ExoQuick™ Isolation. *J. Clin. Med.* 2020, *9*, 1429

**DOI:** 10.3390/jcm10153343

**Published:** 2021-07-29

**Authors:** Timo Gemoll, Sarah Strohkamp, Svitlana Rozanova, Christian Röder, Sonja Hartwig, Holger Kalthoff, Stefan Lehr, Abdou ElSharawy, Jens K. Habermann

**Affiliations:** 1 Section for Translational Surgical Oncology & Biobanking, Department of Surgery, University of Lübeck and University Hospital Schleswig-Holstein, 23562 Lübeck, Germany; strohkamp@hp-hamburg.de (S.S.); sv.rosanova@gmail.com (S.R.); jens.habermann@uni-luebeck.de (J.K.H.); 2 Institute for Experimental Cancer Research, University of Kiel, 24105 Kiel, Germany; c.roeder@email.uni-kiel.de (C.R.); hkalthoff@email.uni-kiel.de (H.K.); 3 Institute for Clinical Biochemistry and Pathobiochemistry, German Diabetes Center at the Heinrich-Heine-University Düsseldorf, Leibniz Center for Diabetes Research, 40225 Düsseldorf, Germany; sonja.hartwig@ddz.uni-duesseldorf.de (S.H.); stefan.lehr@ddz.uni-duesseldorf.de (S.L.); 4 German Center for Diabetes Research (DZD), 85764 München-Neuherberg, Germany; 5 Institute of Clinical Molecular Biology, Center of Molecular Sciences, University of Kiel, 24118 Kiel, Germany; a.sharawy@mucosa.de; 6 Division of Biochemistry, Chemistry Department, Faculty of Sciences, Damietta University, New Damietta City 34511, Egypt; 7 Interdisciplinary Center for Biobanking-Lübeck (ICB-L), University of Lübeck, 23562 Lübeck, Germany

The authors wish to make the following corrections to this paper [1]:

**1.**  **Change in Author Names**

In the original version of our article [1], insufficient acknowledgement was given for the proteomic experiments, sample selection, analysis of results and drafting of the manuscript. We apologize for the original error and any inconvenience caused by the omission of Sarah Strohkamp as an author. To correct this oversight, Sarah Strohkamp has been added as an author including a shared first authorship.

The corrected author list is provided below:

Timo Gemoll ^1,^*^,†^, Sarah Strohkamp ^1,†^, Svitlana Rozanova ^1^, Christian Röder ^2^, Sonja Hartwig ^3,4^, Holger Kalthoff ^2^, Stefan Lehr ^3,4^, Abdou ElSharawy ^5,6^ and Jens K. Habermann ^1,7^

^1^  Section for Translational Surgical Oncology & Biobanking, Department of Surgery, University of Lübeck and University Hospital Schleswig-Holstein, 23562 Lübeck, Germany; strohkamp@hp-hamburg.de (S.S.); sv.rosanova@gmail.com (S.R.); jens.habermann@uni-luebeck.de (J.K.H.)
^2^  Institute for Experimental Cancer Research, University of Kiel, 24105 Kiel, Germany; c.roeder@email.uni-kiel.de (C.R.); hkalthoff@email.uni-kiel.de (H.K.)
^3^  Institute for Clinical Biochemistry and Pathobiochemistry, German Diabetes Center at the Heinrich-Heine-University Düsseldorf, Leibniz Center for Diabetes Research, 40225 Düsseldorf, Germany; sonja.hartwig@ddz.uni-duesseldorf.de (S.H.); stefan.lehr@ddz.uni-duesseldorf.de (S.L.)
^4^  German Center for Diabetes Research (DZD), 85764 München-Neuherberg, Germany
^5^  Institute of Clinical Molecular Biology, Center of Molecular Sciences, University of Kiel, 24118 Kiel, Germany; a.sharawy@mucosa.de
^6^  Division of Biochemistry, Chemistry Department, Faculty of Sciences, Damietta University, New Damietta City 34511, Egypt
^7^  Interdisciplinary Center for Biobanking-Lübeck (ICB-L), University of Lübeck, 23562 Lübeck, Germany

* Correspondence: timo.gemoll@uni-luebeck.de; Tel.: +49-451-3101-8703
^†^  These authors contributed equally to this work.

**2.**  **Change in Main Body Paragraphs**

We have found two inadvertent errors in our paper published in the *Journal of Clinical Medicine* [1].

(1) In the original article, there was a mistake in the legend for Figure 2. Labeling of proteins more greatly expressed in respective groups was inverted. The correct legend appears below. The authors apologize for any inconvenience caused and state that the scientific conclusions are unaffected.

Figure 2. Supervised PCA plots based on 226 (A) and 93 (B) significant spots obtained by ultra-centrifugation or ExoQuick™. Red, proteins with higher expression isolated by ExoQuick™; green, proteins with higher expression isolated via ultracentrifugation.

(2) In the original article, there was a mistake in Supplementary Table S1. The table presents the mean and not the median of the patient cohort. The corrected Supplementary Table S1 was changed accordingly and was included as a separate document. The authors apologize for any inconvenience caused and state that the scientific conclusions are unaffected.

**3.**  **Change in Figures/Tables**

The author wishes to make the following correction to this paper [1]. Due to mislabeling in Figure 2, this figure has to be replaced with the corrected version:

Old Figure 2 with mislabeling:


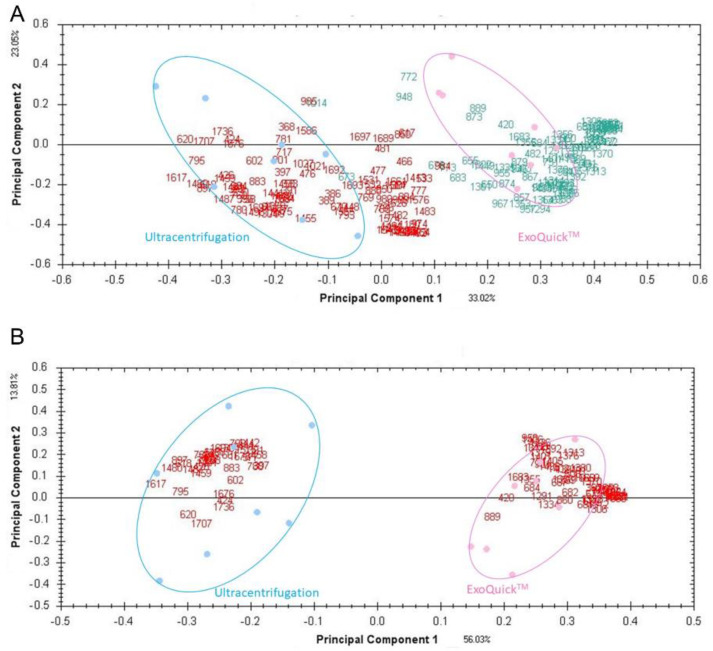


New corrected Figure 2 for replacement: 


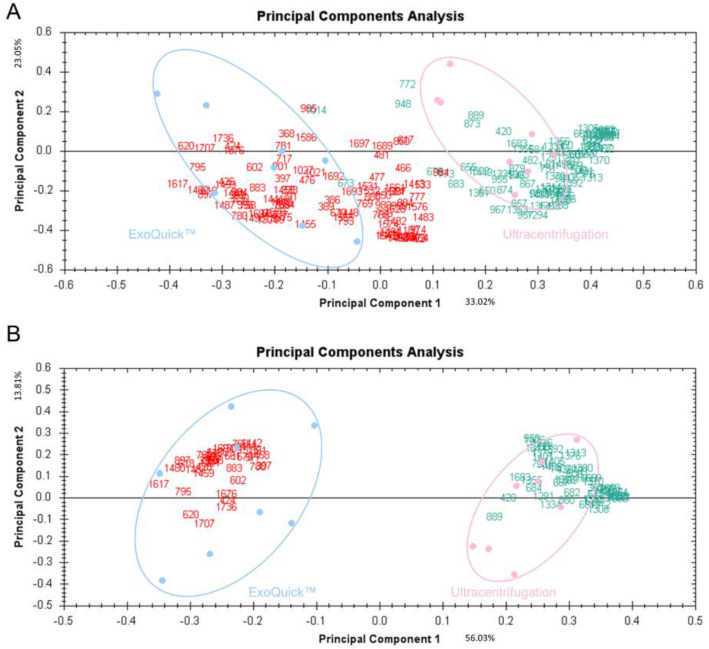

**4.**  **Change in Acknowledgement**

Due to a change in the author list, the Acknowledgement section was deleted from the original article version [1].

**5.**  **Change in the Authors Contributions**

Due to the change in the author list, the Author contributions were corrected in the original article version [1]. 

The corrected author contributions are provided below:

Author Contributions: T.G. designed the study, performed result analysis, and wrote the manuscript, S.S. was involved in experimental design and proteomics experiments, conducted sample selection, performed analysis of results and drafted the manuscript, S.R. was involved in proteomics experiments and data evaluation, C.R. performed sample preparations and revised the manuscript, S.H. performed MALDI-TOF analysis, A.E. designed the study, helped in the selection and preparation of serum EVs, and revised the manuscript, S.L. made substantial contributions to mass spectrometric analysis and wrote the manuscript, H.K. designed the study and wrote the manuscript, J.K.H. designed the study, analysed results, and drafted the manuscript. All authors read as well as approved the final manuscript and agreed to be personally accountable for the author’s own contributions. All authors ensure that questions related to the accuracy or integrity of any part of the work are appropriately investigated, resolved, and the resolution documented in the literature. All authors have read and agreed to the published version of the manuscript.

The authors would like to apologize for any inconvenience caused to the readers by these changes.
