# Supplementary material for: Protein Profiling of Serum Extracellular Vesicles Reveals Qualitative and Quantitative Differences after Differential Ultracentrifugation and ExoQuick™ Isolation
Source: J Clin Med. 2020 May 12;9(5):1429. doi: 10.3390/jcm9051429 (PMC7290673; doi:10.3390/jcm9051429)

**Supplementary Figure S2:** Representative 2-DIGE protein profiles of serum-derived EVs (a-d) and corresponding supernatants (**e,f**). (**a**) EVs isolated using ultracentrifugation (**b**) EVs isolated by polymer-based ExoQuick^TM^ precipitation kit (**c**) EVs isolated using ultracentrifugation and enriched with ProteoMiner^TM^ and (**e**) corresponding supernatant (**d**) EVs isolated using ExoQuick^TM^ and enriched with ProteoMiner^TM^ and (**f**) corresponding supernatant.


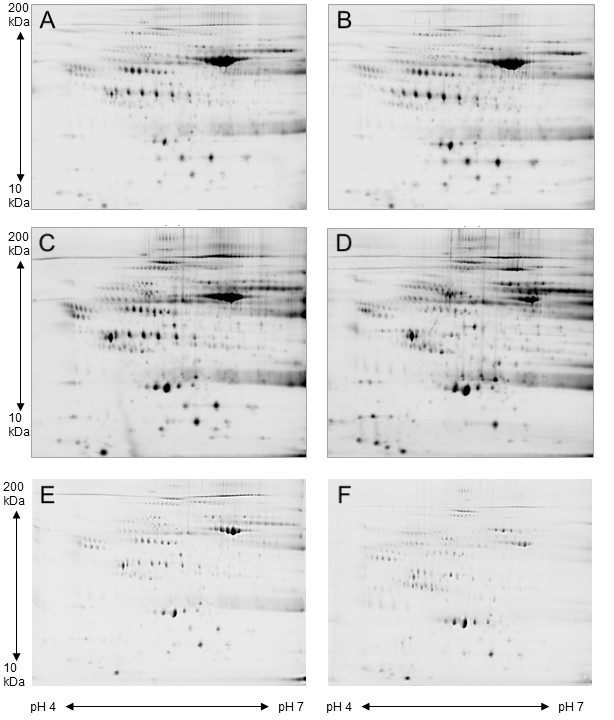

Supplement: Supplementary file 1 [file jcm-09-01429-s001.zip › Supplementary Figure S2.docx]
