# Supplementary material for: Protein Profiling of Serum Extracellular Vesicles Reveals Qualitative and Quantitative Differences after Differential Ultracentrifugation and ExoQuick™ Isolation
Source: J Clin Med. 2020 May 12;9(5):1429. doi: 10.3390/jcm9051429 (PMC7290673; doi:10.3390/jcm9051429)

**Supplementary Figure S3:** Hierarchical cluster analysis of 936 protein spots visualized with dendrograms for both ultracentrifugation and ExoQuick™. Clusters of EV pellets (red) and their corresponding supernatant (green) could be clearly distinguished.


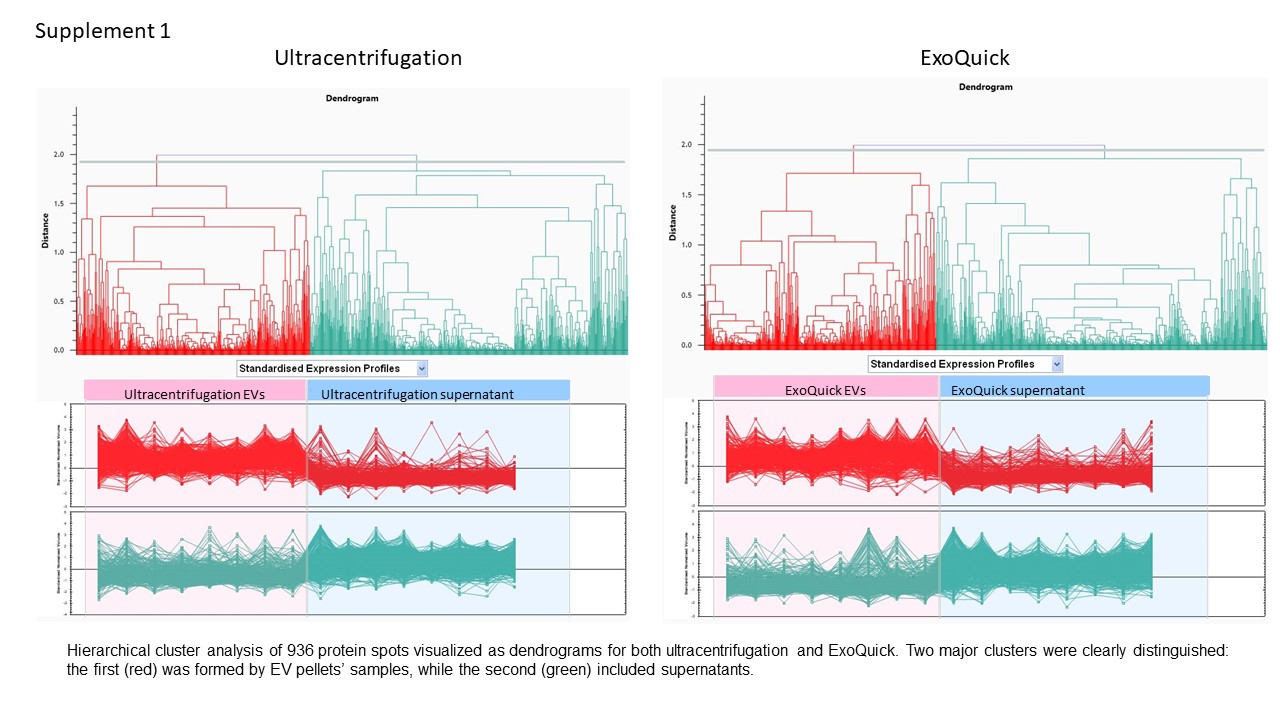

Supplement: Supplementary file 1 [file jcm-09-01429-s001.zip › Supplementary Figure S3.docx]
