# Supplementary material for: Protein Profiling of Serum Extracellular Vesicles Reveals Qualitative and Quantitative Differences after Differential Ultracentrifugation and ExoQuick™ Isolation
Source: J Clin Med. 2020 May 12;9(5):1429. doi: 10.3390/jcm9051429 (PMC7290673; doi:10.3390/jcm9051429)

**Supplementary Figure S4: Western blot validation of Gelsolin in EVs isolated with ultracentrifugation (A) and ExoQuick™ (B).** Specific antibody-targeted protein bands of Gelsolin were detetced by Cy3-labeled secondary antibody. Cy5 total protein signals within each lane were used for normalization (Cy3/Cy5 ratio).


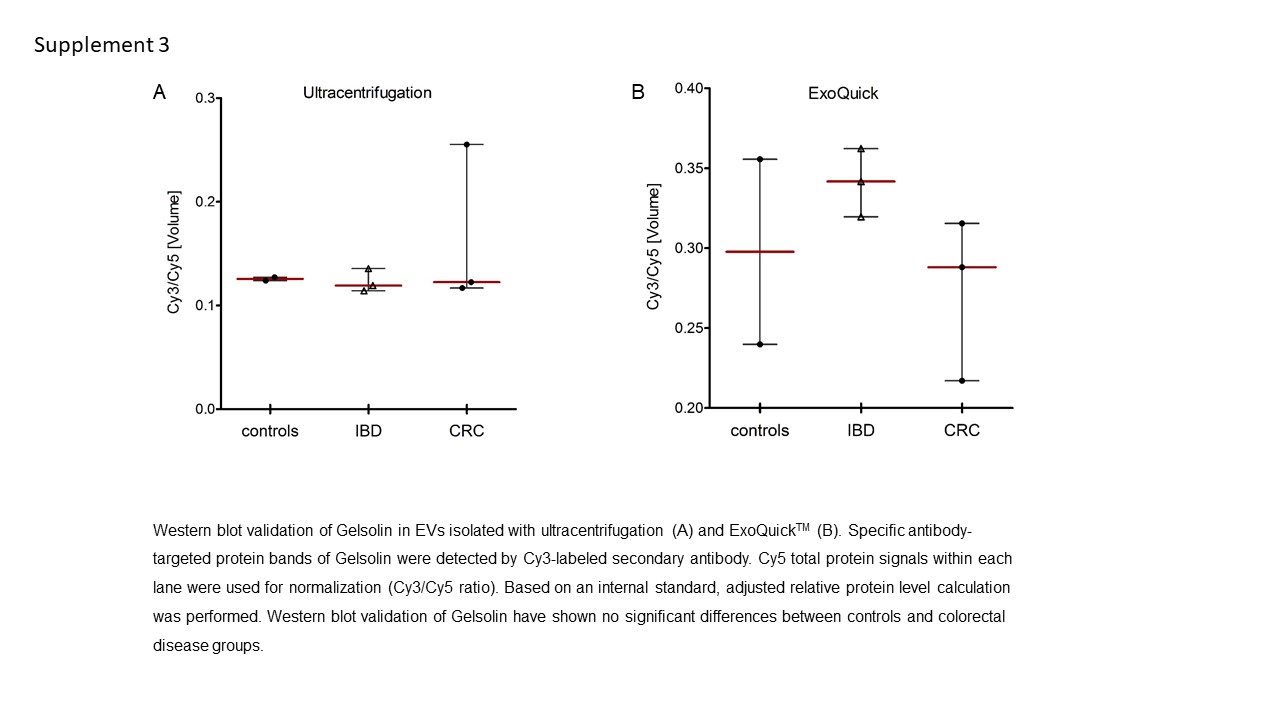

Supplement: Supplementary file 1 [file jcm-09-01429-s001.zip › Supplementary Figure S4.docx]
