# Supplementary material for: Protein Profiling of Serum Extracellular Vesicles Reveals Qualitative and Quantitative Differences after Differential Ultracentrifugation and ExoQuick™ Isolation
Source: J Clin Med. 2020 May 12;9(5):1429. doi: 10.3390/jcm9051429 (PMC7290673; doi:10.3390/jcm9051429)
Supplement: Supplementary file 1 [file jcm-09-01429-s001.zip › Supplementary Table S1.docx]

**Supplementary Table S1.** Patient cohort for 2D-DIGE protein profiling of extracellular vesicles.

|  | Pool no. | Sex | Age at diagnosis | Age at diagnosis | |
| --- | --- | --- | --- | --- | --- |
|  |  |  |  | Median [years] | Range [years] |
| **CRC**  **UICC^1^ stage II** | **1** | M | 67 | 71 | 62 – 77 |
|  |  | M | 62 |  |  |
|  |  | F | 74 |  |  |
|  |  | M | 70 |  |  |
|  |  | F | 74 |  |  |
|  |  | F | 77 |  |  |
|  | 2 | M | 83 | 84 | 82 – 87 |
|  |  | F | 82 |  |  |
|  |  | M | 86 |  |  |
|  |  | F | 87 |  |  |
|  |  | M | 82 |  |  |
|  |  | F | 82 |  |  |
|  | 3 | F | 64 | 59 | 45 – 68 |
|  |  | F | 45 |  |  |
|  |  | F | 68 |  |  |
|  |  | M | 59 |  |  |
|  |  | M | 58 |  |  |
|  |  | M | 62 |  |  |
| IBD  (Ulcerative colitis) | 4 | F | 61 | 63 | 61 – 66 |
|  |  | M | 61 |  |  |
|  |  | F | 66 |  |  |
|  |  | F | 62 |  |  |
|  |  | M | 63 |  |  |
|  |  | M | 65 |  |  |
|  | 5 | F | 18 | 20 | 17 – 22 |
|  |  | F | 20 |  |  |
|  |  | F | 17 |  |  |
|  |  | M | 20 |  |  |
|  |  | M | 22 |  |  |
|  |  | F | 21 |  |  |
|  | 6 | M | 44 | 45 | 42 – 48 |
|  |  | F | 46 |  |  |
|  |  | F | 43 |  |  |
|  |  | M | 45 |  |  |
|  |  | F | 48 |  |  |
|  |  | M | 42 |  |  |
| Clinical control | 7 | M | 45 | 43 | 23 – 64 |
|  |  | F | 23 |  |  |
|  |  | F | 30 |  |  |
|  |  | M | 37 |  |  |
|  |  | M | 64 |  |  |
|  |  | M | 57 |  |  |
| Clinical control | 8 | M | 70 | 53 | 37 – 70 |
|  |  | M | 58 |  |  |
|  |  | M | 48 |  |  |
|  |  | M | 52 |  |  |
|  |  | F | 37 |  |  |
|  |  | M | 54 |  |  |

UICC, Union Internationale Contre le Cancer.
